# Supplementary material for: Cerebrospinal Fluid (CSF) Exchange with Artificial CSF Enriched with Mesenchymal Stem Cell Secretions Ameliorates Experimental Autoimmune Encephalomyelitis
Source: Int J Mol Sci. 2019 Apr 11;20(7):1793. doi: 10.3390/ijms20071793 (PMC6480705; doi:10.3390/ijms20071793)
Supplement: Supplementary file 1 [file ijms-20-01793-s001.pdf]

International Journal of
Molecular Sciences

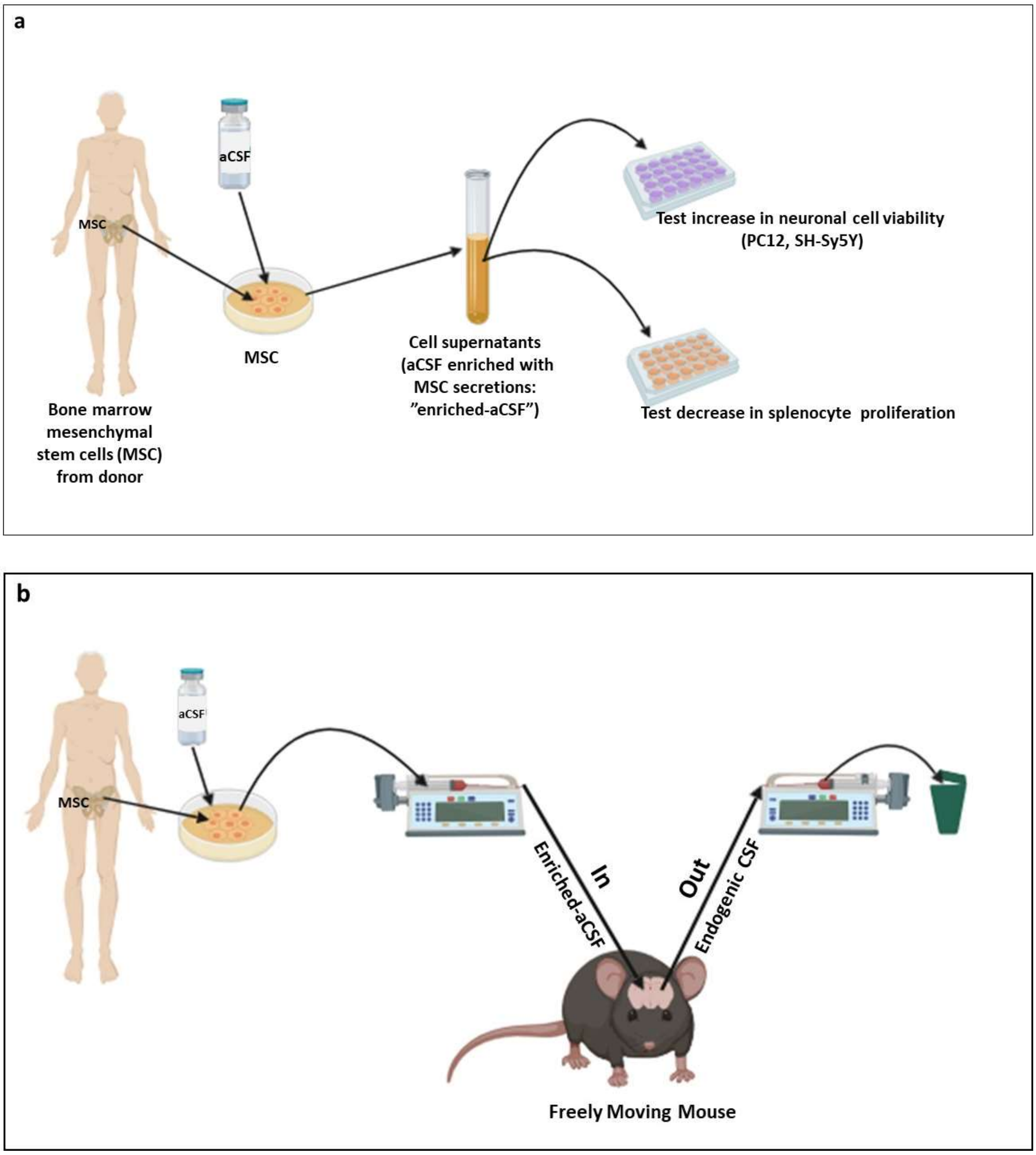

Figure S1. Study design. (**a**) MSCs drawn from bone marrow were grown in artificial CSF (aCSF) and the supernatants collected (and frozen), and further used as "MSC secretions-enriched-aCSF" ("enriched-aCSF") for testing their effect on the viability of neuronal cell lines and proliferation of splenocytes. (**b**) The enriched-aCSF was then used for the CSF exchange therapy in EAE-mice: infusion of the enriched-aCSF into one ventricle ("in"), and withdrawal of endogenous CSF from the other ventricle ("out"), while freely moving in the cage (Explained in the Materials and Methods).
